# Understanding cellular growth strategies via optimal control

**DOI:** 10.1098/rsif.2022.0744

**Published:** 2023-01-04

**Authors:** Tommi Mononen, Teemu Kuosmanen, Johannes Cairns, Ville Mustonen

**Affiliations:** ^1^ Department of Computer Science, Organismal and Evolutionary Biology Research Programme, University of Helsinki, Helsinki 00014, Finland; ^2^ Institute of Biotechnology, University of Helsinki, Helsinki 00014, Finland

**Keywords:** experimental evolution, control theory, optimality, microbes, adaptive traits

## Abstract

Evolutionary prediction and control are increasingly interesting research topics that are expanding to new areas of application. Unravelling and anticipating successful adaptations to different selection pressures becomes crucial when steering rapidly evolving cancer or microbial populations towards a chosen target. Here we introduce and apply a rich theoretical framework of optimal control to understand adaptive use of traits, which in turn allows eco-evolutionarily informed population control. Using adaptive metabolism and microbial experimental evolution as a case study, we show how demographic stochasticity alone can lead to lag time evolution, which appears as an emergent property in our model. We further show that the cycle length used in serial transfer experiments has practical importance as it may cause unintentional selection for specific growth strategies and lag times. Finally, we show how frequency-dependent selection can be incorporated to the state-dependent optimal control framework allowing the modelling of complex eco-evolutionary dynamics. Our study demonstrates the utility of optimal control theory in elucidating organismal adaptations and the intrinsic decision making of cellular communities with high adaptive potential.

## 1. Introduction

Microbial experimental evolution is used for detailed investigation of evolution in laboratory conditions [1]. Following the lead of the long-term evolution experiment of *Escherichia coli* project, spanning more than 70 000 generations, the field has blossomed during the last decade [2]. A typical experimental design assigns selection over a microbial population (or a community), and the population will then evolve under the chosen selection pressure. Clonal microbial populations are inoculated into medium and by a serial transfer protocol at the end of each culture cycle, a fixed proportion is transferred into fresh medium. This procedure continues over a selected number of cycles and the results are contrasted with the wild-type (ancestral) population or control populations propagated under identical laboratory conditions except for lacking the selection pressure of interest.

A trait that increases relative fitness will increase in frequency. However, in general, this can happen together with other traits that might be just incidental or compensatory (or only reveal themselves in different conditions). Traits are often connected with each other through genetic mechanisms, and adaptations cannot, therefore, necessarily occur independently, which is typically optimal. Such evolutionary constraints can slow down or prevent the occurrence of further adaptations [3]. Accepting that the traits themselves evolve under (possibly strong) constraints, we can still ask how these traits could be best used.

Optimal control theory [4] gives us the possibility to test how to optimally use traits, that evolution has already found, in a given environmental context. We can investigate, for example, the benefits of an adaptive use of lower and higher metabolic rates. Microbes can sense population density via specific signalling molecules (quorum sensing) [5,6] or more general environmental cues (e.g. oxidative stress, single-stranded DNA from damaged cells, pH changes and accumulation of metabolic by-products at toxic levels), and react to nutrient deprivation by lowering their metabolic level [7] and entering to stasis [8]. As soon as a target is selected, for example, maximizing population size and environmental conditions are set (e.g. carrying capacity), we can derive the optimal strategy to use a set of given traits. By comparing such optimal solutions to wet laboratory evolution experiments and biological literature, we can assess how close populations are to using the optimal solution, and thus learn about growth strategies and their constraints.

Here, we will use optimal control theory to better understand growth strategies and their evolutionary consequences. We choose to use the traditional Bellman equations and discrete approach instead of commonly used ODE approaches, because we have to capture also small population size dynamics correctly.

Applying optimal control in the context of evolution is a re-emerging field [9], with current focus of optimal control on optimal therapies, directed trait evolution and host–pathogen systems [10–14]. By contrast, our setting here is from the perspective of the intrinsic optimal decision making within a population (see also [15]) in a context typical for laboratory evolution studies. Earlier applications can be found from the field of behavioural ecology, including studies to solve optimal age- and size-dependent life-history strategies [16–18]. Control theory has also been used to study how evolving populations use information and possible modes of transmitting genetic material [19,20], and there is a rising interest on the systems biology side as well [21,22].

We use the already mentioned metabolism example as a case study. We will compute and analyse an optimal solution for a microbe that is able to use two metabolic modes: lower and higher. The aim is to learn how to use these two modes to maximize the population size (yield) under a stochastic logistic growth model. The optimal solution can be seen as the expected outcome of an evolved microbial phenotype that has gone through a serial transfer experiment. Therefore, the phenotype will be adapted to the cycle length used [23] and maximizes population size at the end of a cycle. This type of selection can be introduced in laboratory conditions by establishing *k* parallel single cell populations each having their own compartment. At the end of the cycle, all populations are mixed, and the populations with higher growth become enriched when transferred into fresh media of the next *k* parallel populations [24]. Hence, we hypothesize that this kind of enrichment process can lead to cycle length adaptation. The length of lag phase and change in a population growth rate can make this adaptation process possible [25–27]. Additionally we assume that, for example, limited space, restricted nutrition or metabolic by-products can create a carrying capacity limitation in bacterial populations or communities.

We then change the context from monoculture to head-to-head competitions between different growth strategies. Adaptive phenotypes compete against other non-adaptive phenotypes having only a single metabolic mode. We hypothesize that adaptive phenotypes are the most competitive in these experiments.

## 2. Results

### 2.1. Optimal growth strategy in a monoculture

#### 2.1.1. Monoculture growth model

Life-history strategies in cell populations can be characterized by each strategy’s birth and death rates. Assume that a cell has two alternative metabolic modes, the *defensive* and the *aggressive* mode, with intrinsic birth and death rates (*β*_def_, *δ*_def_) and (*β*_aggr_, *δ*_aggr_), respectively. When using the defensive mode, the cell increases its survival at the expense of proliferation whereas in the aggressive mode the cell invests more of its resources to rapid proliferation, constituting a fundamental life-history trade-off between survival and proliferation. Hence we assume that the intrinsic birth and death rates satisfy the following basic properties:

2.1 $$\begin{array}{rl} & (i)\;\;0<\delta_{\mathrm{def}}<\delta_{\mathrm{aggr}}\\ & (\mathrm{ii})\;\;0<\beta_{\mathrm{def}}-\delta_{\mathrm{def}}<\beta_{\mathrm{aggr}}-\delta_{\mathrm{aggr}}. \end{array}\}$$

The first assumption guarantees that the defensive mode has longer expected lifetime, whereas the second assumption guarantees that the aggressive strategy has a higher intrinsic *per capita* growth rate.

Finite resources necessarily imply the existence of some environmental feedback mechanism which regulates population density. Indeed, let us denote the total population size by *N* and let *K* be the population size at which the *per capita* growth rate becomes zero (the carrying capacity). We model the population growth by a discrete stochastic logistic model, where we assume that the carrying capacity realizes solely by increasing the death rate while allowing the birth rate to remain constant. The effective birth and death rates for different growth strategies are then

2.2 $$\;\begin{array}{rl} \tilde{\beta }(N_{t},t) & =\beta_{m(N_{t},t)}\\ \mathrm{and}\;\;\tilde{\delta }(N_{t},t) & =\delta_{m(N_{t},t)}+(\beta_{m(N_{t},t)}-\delta_{m(N_{t},t)})\frac{N_{t}}{K}, \end{array}\}$$

where *m* : [0, *Kl*] × [0, *T*] → {def, aggr} is the mode which the cell uses at time *t* ∈ [0, *T*] if the population is at size *N*_*t*_ ∈ [0, *Kl*]. The parameter *l* > 1 gives the range in which the strategy is determined above the carrying capacity, which is important to handle the stochastic fluctuations. *Kl* has to be larger than any typically reached population size (*K* + fluctuations), and allows us to make the system computable. Otherwise the state space would extend to arbitrarily large populations with probability of finding a population from the large sizes extremely small.

We discretize the continuous-time stochastic system by drawing the number of births and deaths from the corresponding Poisson distributions at each time step. Each simulation starts from initial population size *N*_initial_ and is updated at the next time step according to the rule

2.3 $$\;\begin{array}{rl} N_{t+1} & =N_{t}+\mathrm{births}(t)-\mathrm{deaths}(t),\\ \mathrm{births}(t) & ∼\mathrm{Poisson}(N_{t}\tilde{\beta }(N_{t},t))\\ \mathrm{and}\;\;\;\mathrm{deaths}(t) & ∼\mathrm{Poisson}(N_{t}\tilde{\delta }(N_{t},t)) \end{array}\}$$

and the simulation proceeds until the chosen end-time *T* is reached. A population goes extinct, if *N*_*t*+1_ ≤ 0 (an absorbing boundary condition). The problem discussed here is to determine the optimal growth strategy *m*_opt_(*N*_*t*_, *t*) for each *t* and *N* which maximizes the expected final population size *N*_*T*_ (yield).

A strategy is called *pure*, if the same mode is used at all conceivable times and population sizes. If different modes are used at different times, we say that the strategy is *adaptive*. If the adaptive strategy is such that at each time point it chooses the mode which maximizes its current *per capita* growth rate, we say that the adaptive strategy is *greedy*. If instead the adaptive strategy restrains to maximize the instantaneous growth at some earlier time points in order to maximize the end population size (the cumulative *per capita* growth rate), we say that the adaptive strategy is *anticipative*. Next we present an optimal control framework which can be used to determine the optimal growth strategies and elucidate the situations where adaptive and anticipative strategies may evolve and replace pure strategies.

#### 2.1.2. Forward propagation of probabilities

In order to compare different growth strategies, we need summary statistics from the process defined in equations (2.2) and (2.3). One approach is to estimate them from averages over individual simulation runs. Alternatively, we can propagate directly the probability distributions of population sizes over time. The change in population size is distributed as

2.4 $$\Delta N(t)∼\mathrm{Skellam}(\mathrm{births}(t)-\mathrm{deaths}(t);\mu_{1},\mu_{2})$$

where $\mu_{1}=N_{t}\tilde{\beta }(N_{t},t)$ and $\mu_{2}=N_{t}\tilde{\delta }(N_{t},t)$. Skellam distributions can be easily computed using convolution of two Poisson distributions. The transition probability from state *N*_*t*_ to state *N*_*t*+1_ using strategy *m*(*N*_*t*_, *t*) can then be written as

2.5 $$W(N_{t+1}|N_{t};m(N_{t},t))=P(N_{t}+\Delta N(t)|N_{t};m(N_{t},t)),$$

and therefore forward propagation of probabilities can be computed as

2.6 $$\;\begin{array}{rl} P(N_{\mathrm{initial}}) & =1\\ \mathrm{and}\;\;P(N_{t+1}) & =\sum_{N_{t}}P(N_{t})W(N_{t+1}|N_{t};m(N_{t},t)). \end{array}\}$$

The Skellam distribution is defined also on the negative side (more deaths than births). Since negative population sizes are biologically unfeasible, we use an absorbing boundary condition at *N*_*t*_ = 0.

#### 2.1.3. Stochastic optimal control solution

We assume that a cell can switch between aggressive and defensive modes without costs, choosing whichever mode is deemed to be more beneficial at that time. Further, only the final population size at the end of the finite time horizon (*t* = *T*) is considered to be important. Such requirement arises naturally during the serial transfer experiments, where the populations are diluted based on their final frequency, but also more generally in biotechnology, where the yield of beneficial bacteria or their metabolites is maximized within a given time-frame (see e.g. [28]). The optimal growth strategy leading to the largest population sizes, can be solved efficiently using the Bellman optimality equation [4].

Formally, we define the expected gain *J*(*N*_*t*_, *t*) describing how large population sizes we can reach in the future if we are in the population size *N*_*t*_ at time *t* and follow the optimal strategy until the end-time is reached. We compare the gains of aggressive and defensive modes at each time point and always select the one that leads to larger population sizes at the end. We can solve the optimal strategy *m*_opt_(*N*_*t*_, *t*) iteratively starting from the end by using a backward-recurrence over all reachable population sizes

2.7 $$\begin{array}{rl} J(N_{t},t) & =\max_{m(N_{t},t)}\sum_{N^{\prime }}J(N^{\prime },t+1)W(N^{\prime }|N_{t};m(N_{t},t))\\ \mathrm{and}\;m_{\mathrm{opt}}\left(N_{t},t\right) & =\underset{m(N_{t},t)}{\mathrm{argmax}}\;J(N_{t},t), \end{array}\}$$

where *T* ≥ *t* ≥ 1 and with a boundary condition *J*(*N*, *T*) = *N* defining our optimization target of maximizing the population size at the end *T*. Hence, this dynamic programming problem can be solved using equations (2.2)–(2.5) and (2.7), together with the boundary condition. Equation (2.7) can also be augmented with other terms that keep track of path rewards or costs at intermediate times and a cost term for use of control (here mode switching). Although including these could be interesting, e.g. to account for resource consumption of the dividing cells, we here focus on the minimal version where only the end population size is important and no cost is assigned to switch of growth mode.

The optimal strategy *m*_opt_ assigns the optimal growth mode for all conceivable states, hence achieving a complete control rule for every possible trajectory. We call this strategy a *control map*.

#### 2.1.4. Control maps for monoculture

First, we investigated the basic behaviour of different growth strategies in a non-competitive setting to form a better understanding of their dynamics. We computed the control maps using the following intrinsic birth and death rates for the defensive and aggressive modes:

**Table:** 

	birth rate (*β*)	death rate (*δ*)	growth rate
aggressive mode	0.125	0.075	0.05
defensive mode	0.025	0.005	0.02

**Figure 1.**
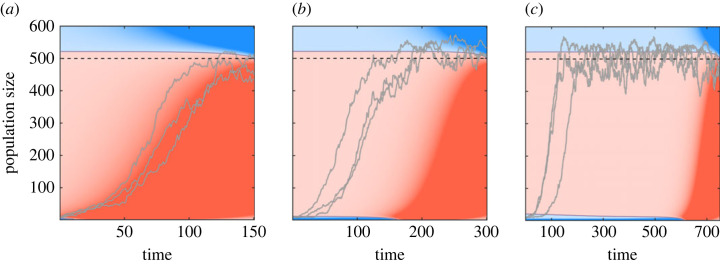
Control maps change qualitatively with respect to the end-time *T*: (*a*) For very short cycle lengths, the optimal growth strategy minimizes the lag and uses the aggressive mode (red shading) even when facing stochastic extinction risk (*T* = 150). (*b*) For longer cycle lengths another defensive mode region appears, where the optimal strategy uses the defensive mode (blue shading at a tiny population region). This creates a distinct lag phase which decreases the stochastic extinction risk (*T* = 300). (*c*) For very long cycle lengths the lag phase becomes even longer up to a point (*T* = 750). Carrying capacity (*K* = 500) is shown with a dashed black line. The intensity of the shading reflects the expected gain of using the optimal mode compared with the alternative.

The pure aggressive strategy leads to much faster growth than the pure defensive strategy, but has also much shorter life expectancy (1/0.075 ≈ 13.33 time units compared with 1/0.005 = 200 time units at initial growth phase). Combined, the average lifetime reproductive success is much higher in the defensive mode than in the aggressive mode (0.025/0.005 = 5 compared with 0.125/0.075 ≈ 1.667). Clearly, the chosen parameter values are to a degree arbitrary, but nevertheless the control maps remain qualitatively similar for all parameters satisfying the basic assumptions, which yield the growth-survival trade-off. Interestingly, we find that it is the cycle length *T* (the growth time before dilution), which changes the control maps qualitatively. For very short cycle lengths, the optimal growth strategy is to minimize the lag phase and grow as aggressively as possible when below the carrying capacity (figure 1*a*). For longer cycle lengths, when the population has enough time to reach the carrying capacity, the optimal strategy starts to use the defensive strategy both at low densities and above carrying capacity (figure 1*b*,*c*). Applying the correct mode becomes increasingly important closer to the time horizon, especially when the system is far from the target. The relative importance of applying the correct mode can be quantified by comparing the expected gains at each point.

#### 2.1.5. Boundaries between two control modes

A boundary (contours separating blue and red regions in figure 1) between the two modes creates a directional flow from the aggressive side (red region) to the defensive side (blue region). The reason for the directional flow is that population size fluctuations are smaller in the defensive mode than in the aggressive mode. Therefore, a big jump from the aggressive side to the defensive side is more likely to occur compared with a reverse jump. Hence we call a boundary *minimizing* if larger population sizes above the threshold boundary use the aggressive mode, as such boundaries create a force that opposes population growth. By contrast, a boundary is *maximizing* if larger population sizes use the defensive mode, in which case the boundary creates a positive force towards larger population sizes.

**Figure 2.**
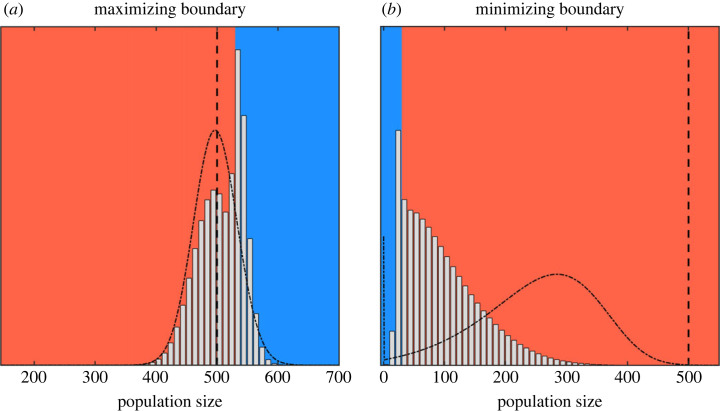
The propagated probability distributions of the optimal strategy (grey bins) compared with the aggressive strategy (dash-dotted line). The maximizing boundary traps the population above the carrying capacity, whereas the minimizing boundary traps the population below the boundary while reducing extinctions. The boundary itself is the reason for the emergence of the lag phase. (*a*) Distribution of the population sizes after *t* = 200 time steps starting from initial population size *N*_initial_ = 500. (*b*) Distribution of the population sizes after *t* = 85 time steps starting from initial population size *N*_initial_ = 10.

Let us look at the two boundaries more carefully (figure 2). The minimizing boundary prolongs the apparent lag phase, slowing down the growth before initiating the exponential growth phase (figure 2*b*). The maximizing boundary on the other hand benefits from population size fluctuations that exceed carrying capacity (figure 2*a*). This boundary essentially tries to trap the population size above the carrying capacity by using the defensive mode whenever the boundary is exceeded (in effect saving resources by using the defensive mode).

The boundaries between the control modes change in time and the anticipative growth strategy (as given by the control map) leverages this time-dependence to maximize the expected yield. The final time slice of the control map (*t* = *T* − 1) corresponds to the greedy strategy, where the maximizing boundary always lies precisely at the carrying capacity. Thus, the length of the time interval becomes irrelevant and the greedy strategy stays constant over time. This corresponds to a simple adaptive strategy predicted by the classical *r*/*K* selection theory, where the aggressive mode is favoured at low densities and the defensive mode at high densities [29].

#### 2.1.6. Cycle length induces selective pressure for lag time evolution

**Figure 3.**
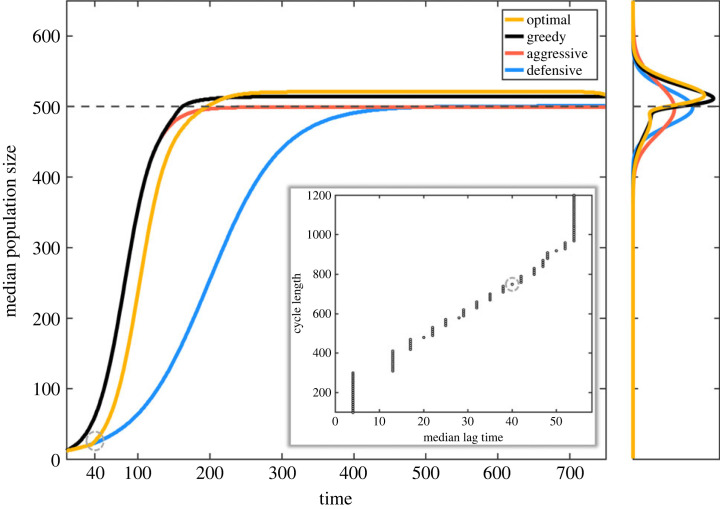
Median population sizes as a function of time for the aggressive, defensive, greedy and optimal (in monoculture) strategies. We note that the adaptive strategies reach higher final population sizes, the optimal marginally beating the greedy strategy. The pure aggressive and defensive strategies have the same median final population size, but the aggressive strategy displays higher variance. The lag time can be defined as the median time when the optimal strategy switches from the defensive to the aggressive strategy (see the dashed circle in the main panel). The embedded panel shows lag times as the function of cycle lengths (the dashed circle matches the cycle length of the main panel). For very short cycles, there is selection to eliminate the lag. For cycles longer than *T* > 300, there appears a distinct lag phase which increases linearly until around *T* = 1000 after which the lag time plateaus.

Next we compare the pure aggressive and defensive strategies with the greedy and the anticipatory growth strategy by propagating the corresponding probability distributions of the population sizes forward in time (figure 3). When the cycle length is sufficiently long, both the aggressive and defensive strategies reach the carrying capacity and produce Gaussian-shaped densities around the carrying capacity. However, since the aggressive strategy has higher growth rate, there is more variance in the final population sizes. Adaptive phenotypes on the other hand can slightly exceed the carrying capacity on average via the efficient switching of the two modes. This is evident by observing the median population sizes (figure 3) and the probability distributions over the final population sizes. The optimal anticipative strategy gains higher yields than the greedy strategy in the monoculture due to its time-dependent control boundaries, which reduce stochastic extinctions and spur aggressive growth also slightly above the carrying capacity.

By defining the apparent lag time as the median time when the optimal strategy switches from the defensive mode to the aggressive mode, we can determine the optimal lag time from the propagated probability distributions as a function of the cycle length. Although the lag time is conventionally defined as the time until the first cell division, in practice the lag time is often determined by fitting slopes to a measured growth curve. Thus, we argue that our definition of lag cannot be distinguished from the observed lag times and that the lag time is an evolvable trait rather than a static or stochastic property [30].

The embedded panel in figure 3 shows the optimal lag time as a function of the cycle length. We note that for very short cycle lengths there is selection to minimize the lag. Then as the cycle length increases, we see that the optimal lag also increases linearly until finally plateauing at very long cycle lengths. This result shows how the used cycle length can generate selective pressure for specific growth strategies with fine-tuned lag times and hence give rise to the optimal, anticipative growth strategy.

### 2.2. Competition under a serial transfer protocol

#### 2.2.1. Biculture competition model

We next investigated the different growth strategies under pairwise (head-to-head) competitions between the anticipative phenotype (monoculture optimal) and the other strategies. We assume that both of the adaptive phenotypes, greedy and anticipative, are able to sense their own, $N_{t}^{a}$, and the competitor’s population sizes, $N_{t}^{c}$, and therefore the community density $N_{t}=N_{t}^{a}+N_{t}^{c}$. However, they either do not have access to any other information regarding the competitor’s behaviour or lack the computational capabilities to use such information systematically to their advantage.

**Figure 4.**
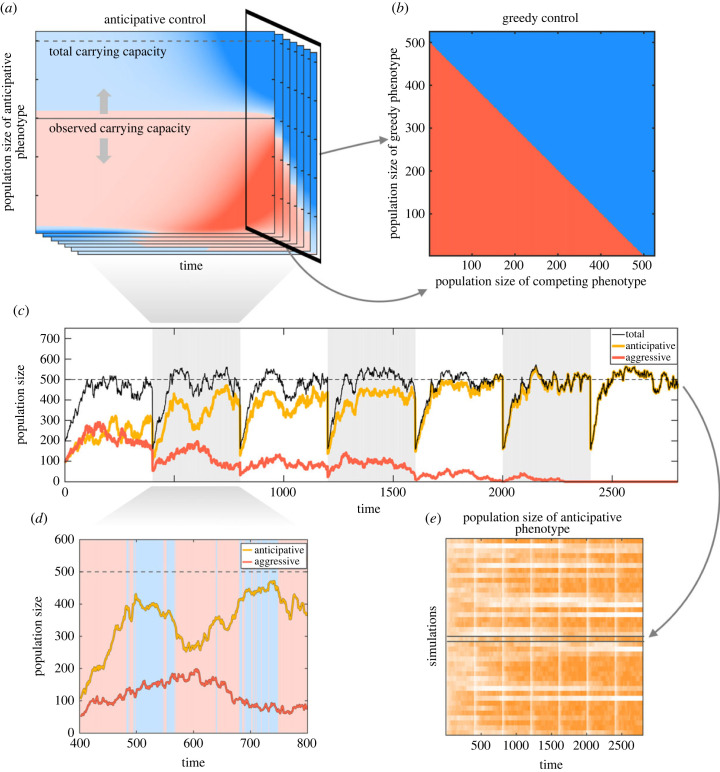
Biculture competition between phenotypes over multiple serial transfers. (*a*) Two phenotypes interact with each other via population densities. The observed carrying capacity by the optimal phenotype changes according to the population size of the competing phenotype. Therefore, the control map is a stack of slices (a control tensor), where the population size of the competing phenotype is fixed for each slice (see text). (*b*) The greedy strategy is an intersecting slice over the last control time point. (*c*) Competition between the anticipative and aggressive phenotypes takes place via mutual carrying capacity. Visible cycles emerge, because at the end of each cycle only a fraction of population (here 20%) is transferred into a fresh media. (*d*) The second cycle is enlarged to show the mode switches of the anticipative phenotype. The aggressive and defensive modes are visualized with red and blue backgrounds. (*e*) A simulation matrix shows 40 runs over time (the example run in (*c*) is emphasized). In most cases only one of the phenotypes survives through all seven cycles (a saturated orange colour denotes a large population size of the anticipative phenotype and the pure white colour its extinction).

The competitor thus has the following death and birth rates:

2.8 $$\begin{array}{rl} \tilde{\beta^{c}}(N_{t}^{c},t) & =\beta_{m(N_{t},t)}\\ \mathrm{and}\;\tilde{\delta^{c}}(N_{t}^{c},t) & =\delta_{m(N_{t},t)}+(\beta_{m(N_{t},t)}-\delta_{m(N_{t},t)})\frac{N_{t}}{K}, \end{array}\}$$

with the *m*(*N*_*t*_, *t*) strategy being either pure (defensive or aggressive) or under greedy control, the mode is defensive if *N*_*t*_ > *K* or aggressive if *N*_*t*_ ≤ *K*. Similarly the anticipative strategy has the following death and birth rates:

2.9 $$\begin{array}{rl} \tilde{\beta^{a}}(N_{t}^{a},N_{t}^{c},t) & =\beta_{m(N_{t}^{a},N_{t}^{c},t)}\\ \mathrm{and}\;\tilde{\delta^{a}}(N_{t}^{a},N_{t}^{c},t) & =\delta_{m(N_{t}^{a},N_{t}^{c},t)}+(\beta_{m(N_{t}^{a},N_{t}^{c},t)}-\delta_{m(N_{t}^{a},N_{t}^{c},t)})\frac{N_{t}}{K}, \end{array}\}$$

with the adaptive strategy $m(N_{t}^{a},N_{t}^{c},t)$ calculated using equation (2.7) as before but now for each $N_{t}^{c}$ value separately. Thus, in our competitive setting, the control map becomes a three-dimensional control tensor (figure 4*a*). At time *t*, the optimal monoculture phenotype uses the control map with a current population size of $N_{t}^{c}$. Therefore, the adaptive strategy can accurately sense the instantaneous carrying capacity $N_{t}/K=N_{t}^{a}/K+N_{t}^{c}/K$ and anticipate its own dynamics in approaching it. But it is not able to factor in the dynamics of the competitor. When competitor’s population size ($N_{t}^{c}$) goes to zero, we recover the monoculture results. Similarly, if the competitor dynamics are slow compared with the time horizon *T*, population size $N_{t}^{c}$ is approximately a constant, and the anticipative strategy is optimal. However, for intermediate cases with fast competitor dynamics the inability for the anticipative strategy to take into account the dynamics of $N_{t}^{c}$ leads to interesting results.

The growth strategy for the greedy phenotype is achieved via a straightforward expansion of the monoculture case. We take a slice over fixed time *t* = *T* − 1 (the last controlled time point) from the control tensor (figure 4*a*,*b*). Instead of a vector (with respect to the monoculture case), we have a control map that does not depend on time but only the population sizes of two phenotypes. The control map for the greedy phenotype is in our model symmetrical, changing its strategy from aggressive to defensive whenever the community density exceeds the carrying capacity.

To demonstrate the setting, we first competed the anticipative (monoculture optimal) and aggressive phenotypes in an *in silico* experimental evolution simulation over several cycles, as shown in figure 4*c*. The anticipative phenotype takes advantage of the two different growth modes to outgrow its competitor (figure 4*d*). In the beginning of each cycle, the community size is diluted to a fixed fraction from where the community starts its new growth (figure 4*c*). In a wet laboratory, this corresponds to taking a sample from a microbial community and transferring it to fresh medium. After several cycles, the stronger competitor is eventually likely to conquer the whole community. Figure 4*e* shows 40 simulation runs with a fixed cycle length, where the aggressive phenotype competes against the anticipative phenotype. Initial population sizes for both phenotypes were set equal. After seven consecutive cycles the anticipative phenotype wins over 26 out of 40 competitions, loses four and in the rest of the simulations both phenotypes coexist. We note that also an inferior phenotype can sometimes win competitions due to random fluctuations.

#### 2.2.2. Cycle length modifies the competitive fitness between the growth strategies

We next demonstrate that the anticipative (monoculture optimal) phenotype makes suboptimal decisions by assuming that the effective, at that time perceived, carrying capacity stays the same.

**Figure 5.**
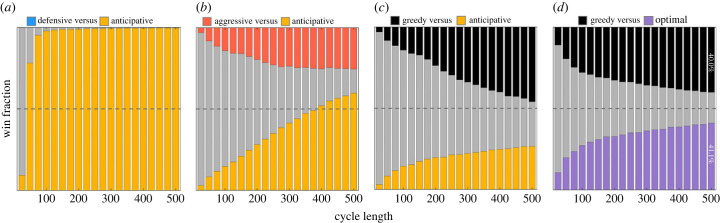
Influence of cycle lengths on pairwise competitions: (*a*–*c*) the results of pairwise competitions (with six cycles) between the anticipative phenotype and the other phenotypes; (*d*) a pairwise competition between the greedy and the biculture optimal phenotype (10 cycles). A grey colour indicates mutual existence of both phenotypes. Dashed horizontal lines denote the 50% win fraction. (*a*) The anticipative phenotype overruns the competition between the defensive and the anticipative phenotype. However, with very short cycles, the defensive populations can mostly persist over a whole run. (*b*) The aggressive phenotype wins a small proportion of competitions against the anticipative, even with long cycles, as it grows faster than the anticipative phenotype in the beginning. Overall, the anticipative phenotype is stronger as it can adapt to changing observed carrying capacity. (*c*) The greedy phenotype combines faster growth and adaptivity. Therefore, it is a stronger competitor than the anticipative phenotype, which is optimized without the knowledge of competitor dynamics. (*d*) The optimal biculture phenotype with the knowledge of competitor dynamics performs just slightly better than the greedy phenotype. The length of competitions (10 cycles) is longer to show the difference between almost equally strong competitors. The panels were computed using *K* = 500 and 20 000 simulations per cycle length. Each new cycle was initiated using a fixed 0.1 K transfer proportion (50 individuals).

The cycle length has a large impact on the relative fitnesses of different phenotypes (figure 5). If the cycle length is too short, populations do not have time to grow and the whole community can end up extinct due to dilutions at the end of cycles. Even if a community survives, all phenotypes using the aggressive growth at low densities—aggressive, greedy and anticipative—are equally fit on average.

Adaptive phenotypes benefit from their two modes only when the cycle length is long enough for communities to reach carrying capacity. Within these intermediate cycle lengths, the adaptive phenotypes start to gain the competitive advantage over non-adaptive phenotypes. The longer the cycle length, the greater the advantage.

The maximizing boundary of the greedy strategy (located at carrying capacity) seems to be more optimal in competition as the anticipative strategy slightly loses to it. The reason is that the community size, which matches carrying capacity is more often reached than community sizes even greater. Therefore, switching later (at larger population sizes) becomes a weaker strategy.

Smaller transfer ratios increase randomness and thereby weaken the relevance of mode switching for survival. In the beginning of each cycle, random fluctuations in population sizes become more important than success in the previous round.

#### 2.2.3. Optimal biculture competitor

Finally, we computed the optimal strategy for the biculture scenario by straightforward extension of equation (2.7) to factor in the full dynamics of the competitor (see electronic supplementary material, S1–S3). The optimal strategy computed this way outperforms the greedy strategy playing competitor as shown in figure 5*d*. Interestingly, when approaching the joint carrying capacity the optimal anticipative strategy pre-emptively switches to using the defensive mode slightly before the carrying capacity is reached (see electronic supplementary material, figure S1). Therefore, the actual change between monoculture and biculture optimal strategies could be approximated by changing the location of the maximizing boundary to be somewhat below the carrying capacity in biculture. This simplicity leaves room for a genetically hard-coded rule to implement a winning control map with only sensing of the carrying capacity necessary and not requiring sophisticated computation as given by electronic supplementary material, equations S1–S3.

## 3. Discussion

In this study, we showed how optimal control theory provides a powerful methodology to assess optimal growth (life history) strategies in different settings and how they could be realized via evolution. We demonstrated in our adaptive metabolism case study how the ability to switch between aggressive and defensive growth modes can help monocultures reach higher yields and drive non-adaptive strains extinct. The adaptive phenotypes won competitions simply by exploiting the interplay of demographic stochasticity and the carrying capacity. Furthermore, we noted how the used cycle length of the serial transfer experiments has practical importance, as it may cause unintentional selection and lag time evolution. The experiments are likely to enrich strains fine-tuned for the specific time horizon.

These examples show how the study of rapid short-term evolution, often driven by human intervention, requires handling of stochastic population dynamics and an explicit notion of time (e.g. time-dependent interventions in drug therapies) to understand how populations can and will respond. However, it has also been argued that evolutionary processes are not always—and in every turn—optimizing the fitness [31]. Despite the fact that responses to selection can be seen as one type of optimization, this must be kept in mind.

In laboratory evolution experiments, microbial populations have been exposed, for example, to starvation and other harsh environments, predation, competition, environmental and temporal fluctuations and time pressure [32–36]. After an experiment, an evolved population will be investigated to reveal the genetic and phenotypic changes that have accumulated under the chosen selection regime. We can test in a shared environment how well the evolved microbes fare in a community consisting of wild-type microbes or other evolved phenotypes. These other phenotypes can introduce additional selective factors, which make the monoculture-evolved phenotype maladapted to the community [37–39]. This may cause the evolved microbes to be lost from the community [40], which is why we included the competition aspect into this paper.

The calculated control maps illuminate the optimal use of traits, giving rise to adaptive life-history strategies. They show how the optimal anticipative strategy with time-dependent control boundaries converges to the greedy strategy at the end of the time horizon. The greedy strategy itself can be (in the simple model considered) viewed as the optimal adaptive strategy at the deterministic limit. Hence, the control maps elegantly show the value of anticipation in any given model. Here we showed how anticipating demographic stochasticity and the limits of growth (carrying capacity) can prove to be useful, but certainly the rewards of anticipation can be much higher. For example, the adaptive immune system can be seen to effectively anticipate reinfection by the already seen pathogen [12].

Adaptive strategies require constant monitoring of environmental cues (such as quorum sensing in bacteria) and hence at least some sort of sensory system, which consumes energy (incurring a fitness cost). Anticipative strategies require even more sophisticated information processing. Hence, adaptive and anticipative strategies must have a substantial advantage over pure strategies to compensate the necessary investments in maintaining such systems [41,42]. Alternatively, the corresponding control maps must be simple enough to be approximately executed by some rule which is hard-coded genetically [12]. Or accessible by a heterogeneous population executing a mixed strategy based on the coexistence of pure strategies in a population as a form of bet-hedging. Interestingly, a recent study focusing on cellular decision making under volatile stochastic environments used methods from mathematical finance, as well as optimal control, to analyse how volatility affects the success of persister phenotypes [15].

We hypothesize that the intrinsic lag phase observed in bacterial growth may be an example of such a hard-coded rule, which can efficiently reduce the risk of stochastic extinctions. While the lag phase may serve also many other important functions which enable future proliferation, it is nevertheless worthwhile to note how the tendency for considerable lag phase arises in our model purely from demographic stochasticity alone. Given the observed variation in microbial lag times and the widespread potential for lag time evolution [30,43,44], our minimal model provides a simple explanation for why we observe distinct lag phases during microbial growth in the first place. Indeed, playing the defensive mode first rather than jumping directly to the aggressive mode may be highly advantageous for the establishment of new mutants and in the context of dispersal to new environments. Thus, the lag phase could be optimized in conjunction with selection of other beneficial traits, which may, therefore, either promote or constrain its evolvability [45].

One particularly interesting direction for future research would be to further investigate the extent of anticipative strategies in biology: are biological algorithms predominantly greedy and when can we expect anticipative behaviour to evolve? As our case study suggests, seemingly anticipative behaviour may emerge simply by the process of natural selection and adaptation to the prevailing environment. However, as our biculture competition model suggests, such optimal monocultures may be easily invaded by a greedy strategy (which may decrease the resource efficiency of the community).

Incorporating more complex and general environmental feedback and resource dynamics to our modelling framework will certainly allow for important insights. In these cases, it may be necessary to adopt other mathematical tools than the Bellman equations [21], if computational burden becomes otherwise too large. Therapies against cancer cell, microbial, pest and parasite populations can all be seen via the lens of systematic growth manipulation. Our setting here could be useful in that context in better understanding the optimal response of the target population to these interventions. Going beyond our case study, we also envision how the control theoretic approach could be highly useful also more generally to test various evolutionary hypotheses, elucidate organismal adaptations, predict likely evolutionary trajectories and steer evolution to a desired direction by manipulating the evolutionary drivers of the traits in question.

## Data Availability

All code used in this study are available via GitHub: https://github.com/RipariaRiparia/optimalgrowth/. Additional information is provided in the electronic supplementary material [46].
